# Case Report: Two Cases of Metastatic Pancreatoblastoma in Adults: Efficacy of Folfirinox and Implication of the Wnt/β-Catenin Pathway in Genomic Analysis

**DOI:** 10.3389/fonc.2021.564506

**Published:** 2021-03-16

**Authors:** Jean-Luc Raoul, Sandrine Oziel-Taieb, Thierry Lecomte, José Adelaide, Arnaud Guille, Max Chaffanet, Flora Poizat, Marie-Françoise Heymann, Louise Barbier, François Bertucci

**Affiliations:** ^1^Department of Medical Oncology, Institut de Cancérologie de l'Ouest, Saint-Herblain, France; ^2^Department of Medical Oncology, Institut Paoli-Calmettes, Marseille, France; ^3^Department of Hepatogastroenterology, CHU Tours, Tours, France; ^4^Predictive Oncology Laboratory, Centre de Recherche en Cancérologie de Marseille, Marseille, France; ^5^Department of Pathology, Institut Paoli-Calmettes, Marseille, France; ^6^Department of Pathology, Institut de Cancérologie de l'Ouest, Saint-Herblain, France; ^7^Department of Digestive Surgery, CHU Tours, Tours, France

**Keywords:** pancreatoblastoma, adult, genomics, chemotherapy—oncology, Wnt/β-catenin

## Abstract

Pancreatoblastomas are unfrequent tumors usually found in children. We report two cases of metastatic pancreatoblastomas observed in young women. A systemic chemotherapy (FOLFIRINOX regimen) was associated with a disease control in one case and a partial response in the second with an improvement of general status for both. A high-throughput sequencing of the tumor described in both cases alteration in the Wnt/β-catenin pathway: a mutation in *CTNNB1* (exon 3, c.110C>G, p.S37C, reported as a hotspot in COSMIC) in one case and a homozygous loss associated with breakage targeting *APC* (5q22.2) in the second.

## Introduction

Pancreatoblastoma is a very rare malignant tumor of the pancreas, usually found in children <10 year-old (1). Less than 50 cases in adults have been reported in the literature (2–4), and their main clinical features are unspecific. Most adult patients are young or very young. They can have pain, weight loss, abdominal mass, or jaundice. Liver metastases are reported in <30% of the cases despite a primary size at diagnosis usually over 10 cm. The diagnosis is based on histopathology. Surgery, including removal of metastases, is the best therapeutic option allowing cures. Efficacy of systemic chemotherapy, particularly platinum salts and doxorubicin, can be impressive in children (5). These tumors seem to be more aggressive in adults than in children, but less aggressive than classical pancreatic ductal adenocarcinoma. Here, we report on two cases of young women diagnosed with a metastatic pancreatoblastoma, successfully treated by a FOLFIRINOX regimen and with alteration of the Wnt/β-catenin pathway identified by high-throughput sequencing of the tumor.

## Case Report n°1

**Figure 1:**
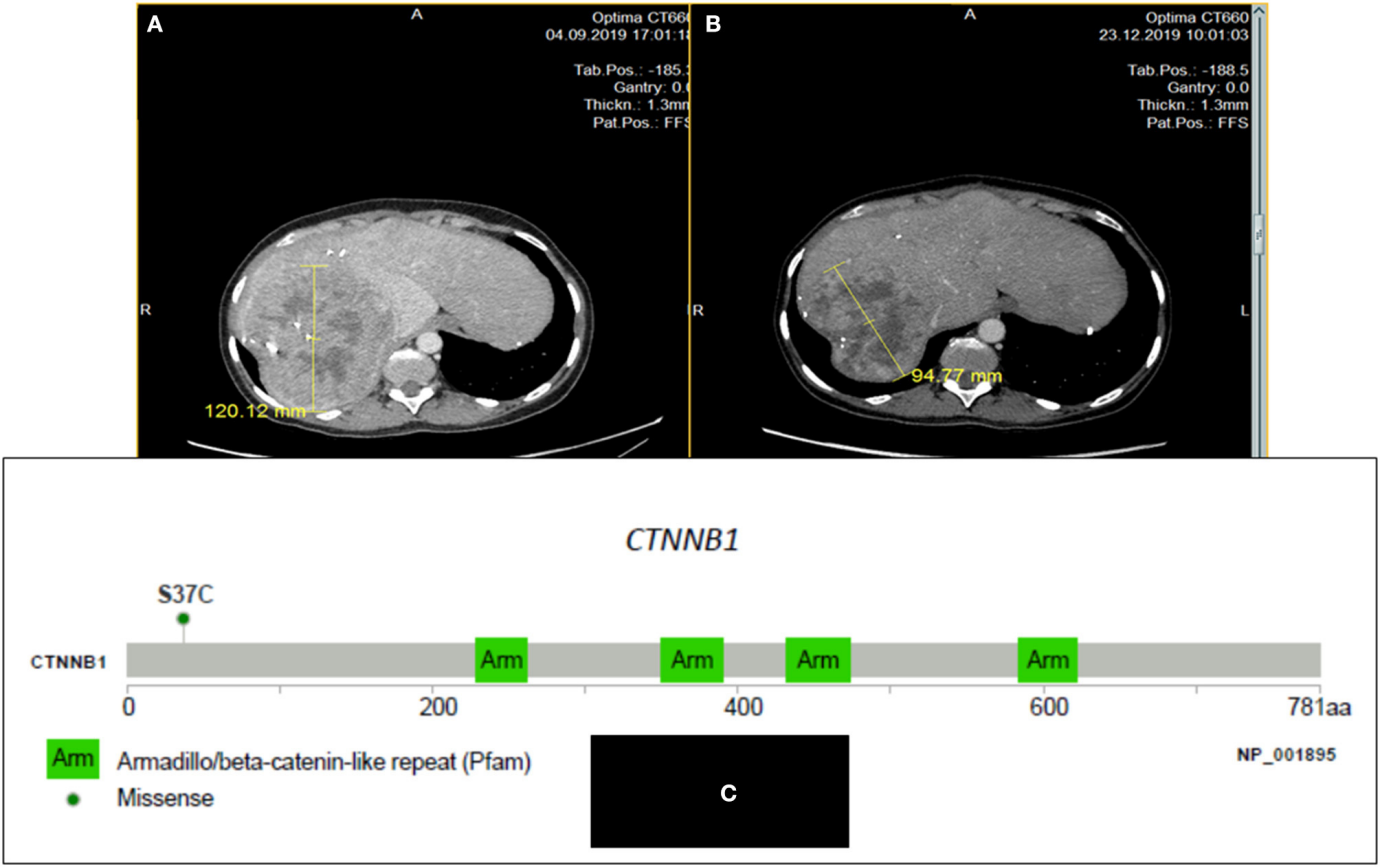
CT scan images of the large liver metastasis of a pancreatoblastoma (patient # 1) in a 33 year-old woman: before **(A)** and after six cycles **(B)** of FOLFIRINOX regimen: minor response; **(C)**: Lolliplot of CTNNB1 gene showing the S37C mutation in exon 3.

This 33-year-old woman, with no prior familial or personal medical history, presented in 2009 epigastric pain and a palpable left upper abdomen quadrant mass. The diagnosis of large pancreatic tumor invading the gastric wall leads to perform a radical R0 distal pancreatectomy. On histopathological examination, the diagnosis of pancreatoblastoma was done in front of an encapsulated mass, with epithelial components with acini, some tubular structures and squamoid corpuscles. No adjuvant treatment was delivered. The follow-up was uneventful until 2012, when three liver metastases were found. Resection and external beam irradiation (Cyberknife®) of metastases were then performed. Liver metastases recurred a few months later (two in the left lobe and a large one in the remnant right lobe) but progressed at a very slow pace and were only submitted to radiological monitoring until October 2019. Then, a clear progression was observed on the large metastasis (Figure 1A) of the right lobe and associated with weight loss and pain, and an increase of serum Alkaline Phosphatases (1.6 ULN); CEA was normal and CA19-9 slightly increased (56 U/mL, normal <39 U/mL), as well as AFP (20.8 ng/mL; N <7 ng/mL). A systemic chemotherapy based on the classical FOLFIRINOX regimen (6) was proposed. Six cycles were delivered from October to December 2019. Tolerance was mild (grade 2 asthenia, and grade 2 nausea despite symptomatic treatments) and no clinical benefit was immediately noticed. Biologically, Alkaline Phosphatase and CA19-9 went back to normal values. On contrast-enhanced CT-scan, we observed a minor response for the main metastasis (from 120 to 95 mm; Figure 1B), and stabilization for the two other ones, thus defining a RECIST 1.1 stable disease. A follow-up was initiated. After 6 months of therapeutic holidays, her general status improved and was by far better than before chemotherapy; the CT-scan remained stable until in mid-December 2020.

In 2014, in order to find out possibilities for targeted therapy, immunohistochemistry (IHC) of the primary tumor searching for ALK, CMET, and ROS-1 was negative as well as the search for gene mutations in *ALK* exons 23, 24, and 25, *MET* exons 14, 16, 17, and 18, and *BRAF* exon 15. On pathological exam, tumor cells displayed a strong cytoplasmic and nuclear staining for β-catenin by IHC. In 2019, after obtaining written informed consent from the patient, a NGS analysis (FOUNDATION ONE® CDx) from DNA extracted from one of the resected liver metastases was performed before initiating chemotherapy. The tumor was classified as MicroSatellite Stable (MSS), the Tumor Mutational Burden (TMB) was low (1 mutation/Mb), and only two somatic gene mutations were retained: *CTNNB1* (exon 3 c.110C>G, p.S37C; Figure 1C) and *NSD3* /*WHSC1L1* (NM 017778.2 exon 1c.121G>A, pA41T); these two mutations are hotspot mutations reported in COSMIC database (respectively COSM5679 and COSM750462). Some variants of unknown significance were also noted: *DIS3* (P666L), *DOT1L* (G724S and R544K), *FGF19* (S147T), *KDR* (R541G), *MYCL1* (S26R), *PTEN* (S294R), and *SPEN* (D1701E). No other base substitution, insertion/deletion, gene fusion, and copy number alteration was observed.

## Case Report n°2

**Figure 2:**
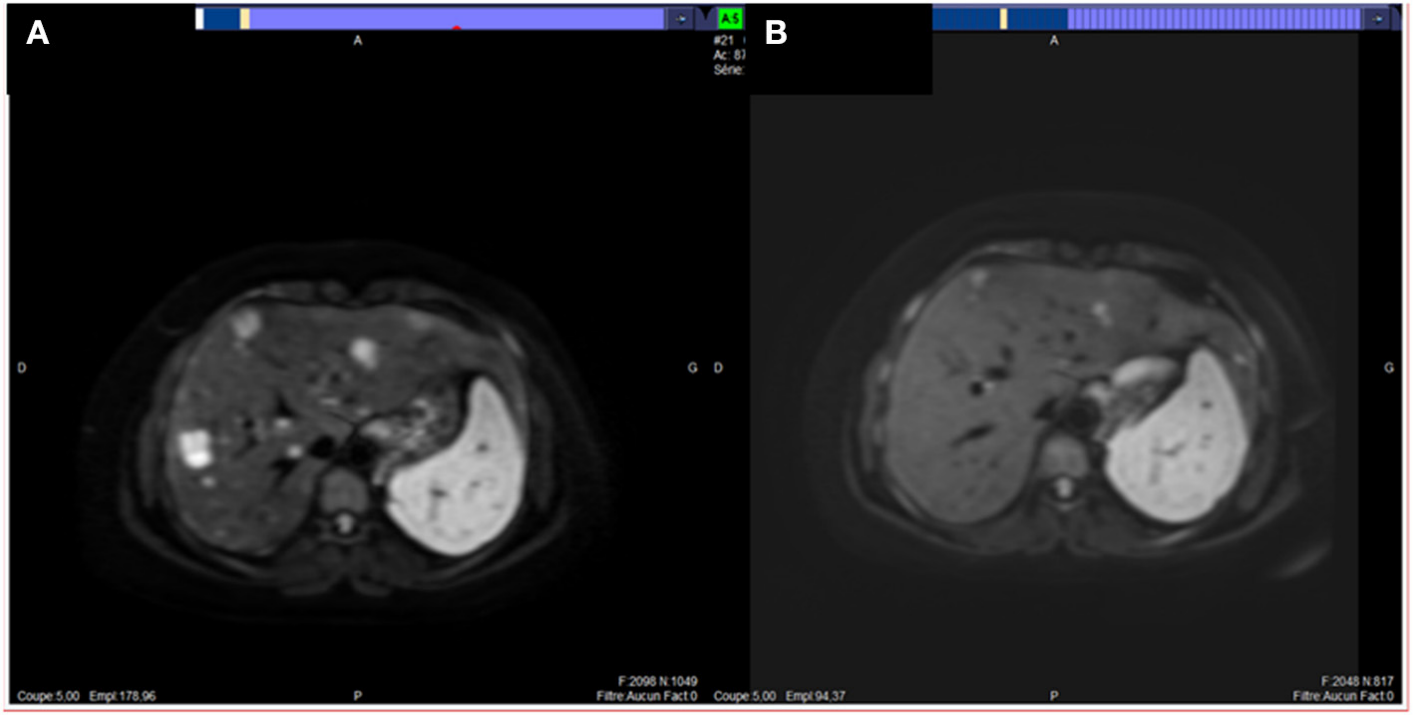
MRI images of liver metastases of our second case of pancreatoblastoma (patient # 2) before **(A)** and after four cycles **(B)** of FOLFIRINOX regimen: partial response.

A 33-year-old woman, with no familial medical history, presented on summer 2019, polyarthralgia and epigastric discomfort with no deterioration of clinical status. A cholecystectomy had been performed in April 2019 for gallbladder stones without any hepatic ultrasound abnormality. A CT-scan, performed as part of the assessment of a possible rheumatismal disease, showed a pancreatic tail tumor associated with multiple liver metastases confirmed by an abdominal MRI (Figure 2A). No thoracic lesion was found. Pathological examination of pancreatic and liver EUS-guided biopsies revealed a very heterogeneous tumor cell population with small cells associated with epithelioid cells; tumor cells displayed a strong cytoplasmic and nuclear staining for β-catenin by IHC. The retained diagnosis was pancreatoblastoma. Pancreatic lesion and liver metastases were hypermetabolic on ^18^FDG-PET. The serum levels of CEA, CA19-9, Chromogranin A, AFP, and lipasemia were normal. Only NSE was slightly increased (36.2 μg/l, normal <16.3 μg/l) as well as γ-GT (6xN). Four cycles of FOLFIRINOX regimen were delivered from February to April 2020. As side effects the patient presented an asthenia (grade 2) and a thrombopenia (grade 3 then 2), requiring dose reduction and delays. CT-scan and hepatic MRI performed after the fourth cycle showed a RECIST 1.1 partial response with a decrease of the maximal diameter of the pancreatic lesion from 44 to 23 mm and a decrease in number and size of all hepatic lesions that became infracentimetric (Figure 2B). Four additionally cycles of FOLFIRINOX were proposed, followed by capecitabine only. After three cycles of capecitabine a progression was seen on the CT-scan and FOLFIRINOX was resumed, beginning mid December 2020. No genetic counceling was proposed.

**Figure 3:**
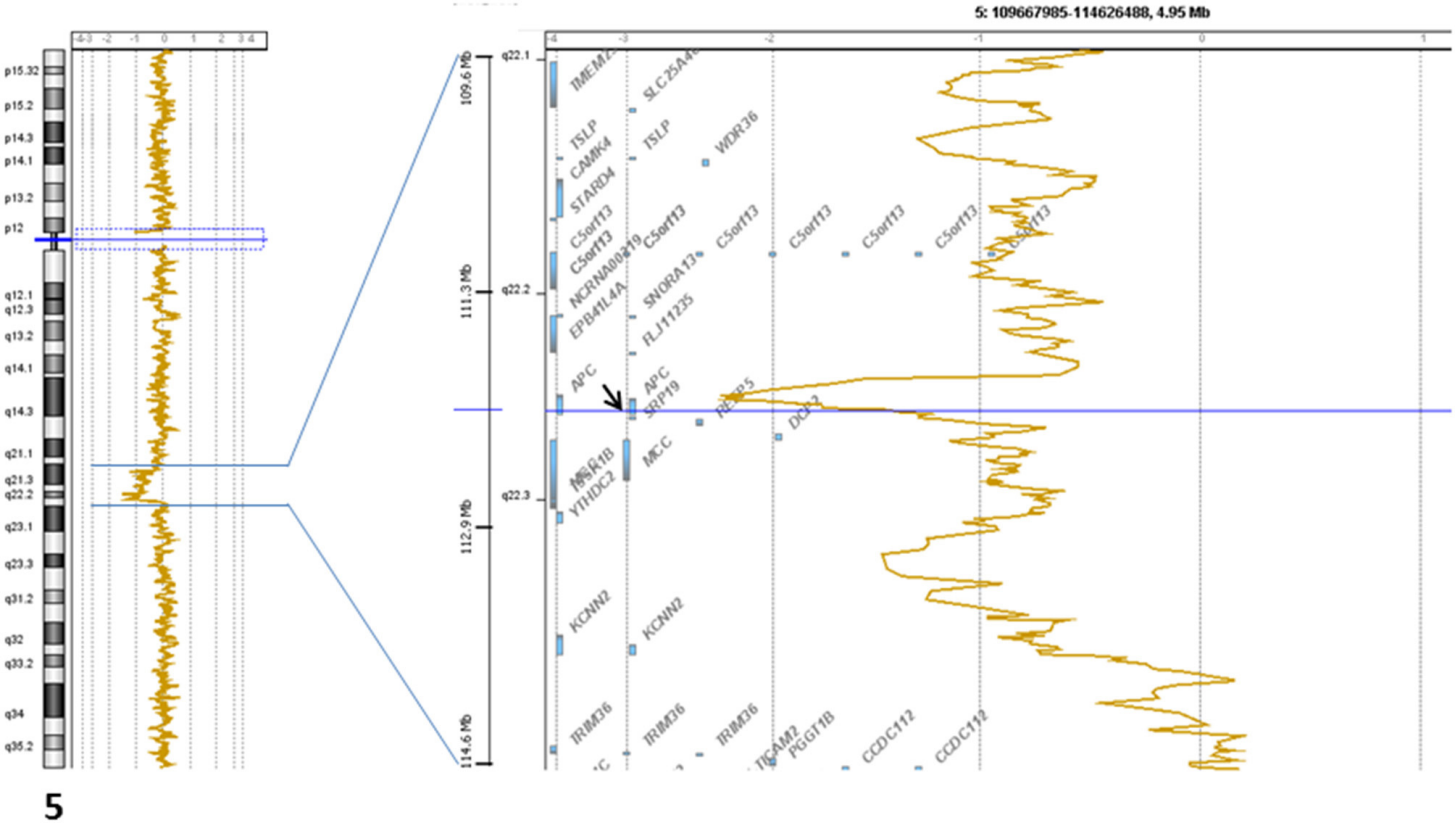
Genomic profile of the 5q22.2 chromosomal region showing the breakage in *APC* gene.

The signed informed consent from the patient was obtained for genomic profiling. DNA extracted from the biopsied primary tumor (and normal blood) was submitted to NGS analysis (institutional Impact V1 panel including 754 genes) and array-CGH (Agilent 4x180k) as previously described (7). The lesion was classified as MSS, and the TMB was low (<1 mutation/Mb). No gene fusion was identified. A deletion associated with a breakage targeting *APC* (5q22.2: Figure 3) was observed. We also found a heterozygous loss of the 9p21 region including *CDKN2A* and the 13q14.2 region including *RB1*, and a focal amplification in the 6p22 region including many histones genes. No pathogenic somatic mutation was identified.

## Discussion

Pancreatoblastoma is a very rare tumor, typically found in young children and originating from fetal pancreatic stem cells. Some cases are associated with genetic syndromes (8), such as the Beckwith-Wiedermann syndrome (mainly caused by genetic or epigenetic defects within the chromosome 11p15.5 region, containing genes like *IGF2* and *CDKN1C*) (9), and Familial Adenomatous Polyposis (*APC* gene) (10). At diagnosis, the median patients' age is 5 years and <20% had metastases, most often hepatic. In adults, the clinical presentation is usually in line with a large pancreatic malignant mass in a young adult with abdominal pain, jaundice, weight loss, and liver nodules. On imaging, these tumors are large, located in the pancreas head in 50% of cases, and have a mixed solid and cystic pattern; imaging can also show some aggressive patterns like invasion of adjacent structure or metastases at diagnosis. Pathologically, these tumors are considered as part of the “pancreatic carcinomas with acinar differentiation,” with acinar cell carcinomas and carcinomas of mixed differentiation (11). Squamoid nests are the key morphologic feature of pancreatoblastoma; a neuroendocrine component is frequently found (12). On IHC, a very distinctive feature is the abnormal nuclear and cytoplasmic localization of β-catenin, usually restricted to the squamoid nests (12).

Surgery of the primary tumor but also of the liver metastases is the first therapeutic option, but recurrences are frequent, and second surgery can be performed. When surgery is not possible, some chemotherapy regimens have been proposed. In our two patients, the FOLFIRINOX regimen, gold standard in pancreatic adenocarcinomas, allowed some improvement after respectively 6 and 4 cycles, with significant decrease in tumor size and improvement in the patients' performance status. As cases are scarce only a few reports with new systemic chemotherapy regimen has been published, but similar response has been reported in one case in the literature (4). The prognosis of pancreatoblastoma is better than that of other pancreatic malignancies with a median overall survival of 48 months. The main prognostic factors are metastases, surgical resectability and age (1). The development of effective systemic therapies is crucial, but the molecular pathogenesis of this rare disease remains poorly known.

During the last years, several studies aimed at defining the molecular landscape of pancreatoblastoma were performed, allowing to improve our knowledge. Clearly, pancreatoblastoma, like other pancreatic carcinomas with acinar differentiation and other rare malignant pancreatic tumors, has a molecular profile different of pancreatic ductal adenocarcinoma, and display frequent alterations of genes involved in the Wnt/β-catenin pathway, notably *CTNNB1* exon 3 mutations. Such mutations are rare in cancers other than liver, uterine, and adrenocortical carcinomas (13). Among a series of 328 pancreatic tumors, only 12 (3.7%) had *CTNNB1* exon 3 mutation, including eight solid pseudopapillary neoplasms, two pancreatic adenocarcinomas, and two pancreatoblastomas (cBioPortal database). In the pancreatic ductal adenocarcinoma, gene alterations are quite different: the most frequent ones concern *KRAS* (95% of the cases), *CDKN2A* (>90%), *TP53* (75%), and *SMAD4* (55%) (14), while *CTNNB1* mutations are very rare (1%) and only found in *KRAS* wild-type tumors (15).

The rare data reported in pancreatoblastoma, suggest similar and important role of the Wnt signaling pathway. On 2001, Abraham et al. (10) reported a series of nine cases, including seven pediatric cases and two adult cases: a nuclear accumulation of β-catenin was observed in 7/9 cases and a molecular alteration of *APC*/β-catenin pathway was observed in 6/9 cases (five activating *CTNNB1* exon 3 mutations and one biallelic *APC* inactivation). On 2003, Tanaka et al. reported a series of seven pediatric cases (16): a nuclear accumulation of β-catenin was observed in all cases and an activating *CTNNB1* mutation (exon 3) was found in 2/5 cases tested. On 2012, one case of congenital pancreatoblastoma in a 3-day-old child was reported with nuclear/cytoplasmic accumulation of β-catenin and an activating *CTNNB1* mutation in exon 3 (17). An integrated molecular analysis of 10 pediatric patients (median age of 3 years) revealed a very high frequency of activation of the Wnt signaling pathway, either *via* somatic mutations of *CTNNB1* (90%) or copy-neutral loss of heterozygoty of *APC* (10%), with concurrent imprinting dysregulation of IGF2 (8). Here too, all *CTNNB1* mutations were observed in exon 3.

On 2018, a somatic heterozygous missense mutation in *APC*, associated with the same heterozygous germline mutation, was reported in a 37-year-old woman with pancreatoblastoma (18). Functional analysis suggested this mutant *APC* attenuated repression of Wnt/β-catenin signaling activity, and was likely involved in the onset of disease. More recently, in a German series of four adult patients with metastatic disease (4), two (out of the three who had whole exome sequencing) had *CTNNB1* exon 3 mutations. Thus, out of the 23 pediatric samples sequenced through six studies, 16 displayed an activating *CTNNB1* mutation in exon 3 and one an inactivating *APC* alteration. Out of the eight adult samples sequenced through these six studies and our two present new cases, four displayed an activating *CTNNB1* mutation in exon 3 and three an inactivating *APC* alteration. Of note, in all cases, *APC* and *CTNNB1* alterations were mutually exclusive, and were associated, when analyzed, with nuclear accumulation of β-catenin.

**Figure 4:**
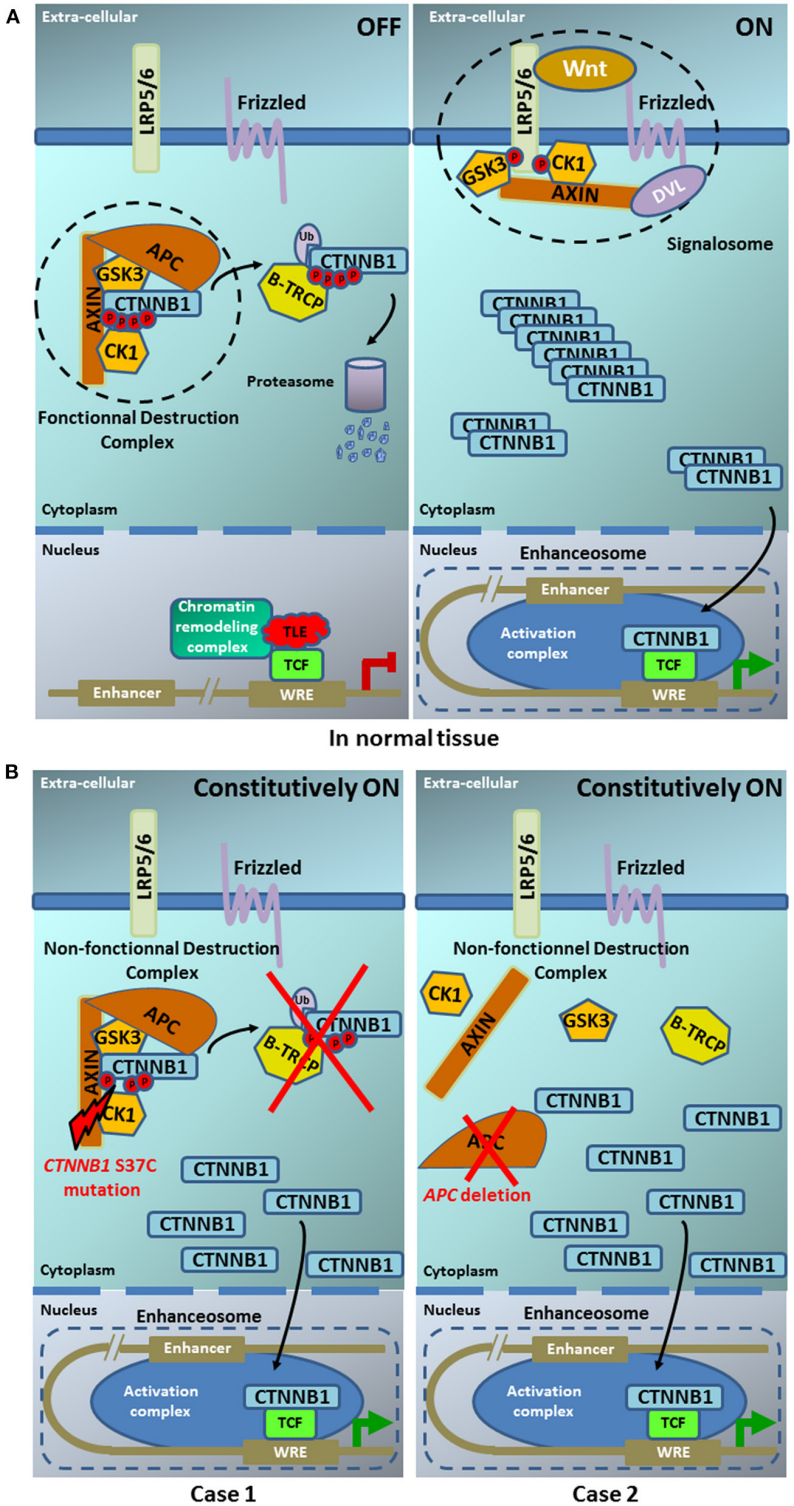
Model of the Wnt pathway in normal tissue **(A)** and in our two pancreoblastoma cases **(B)**. **(A)** In normal tissue— (left part) ln the absence of Wnt ligand, the β-catenin destruction complex maintains low cytoplasmic levels of β-catenin. In the destruction complex, β-catenin is phosphorylated at sites S33/S37 and T41/S45 by casein kinase 1 (CK1) and glycogen synthase kinase 3 (GSK3), respectively. Phosphorylated β-catenin is recognized by the β-Transducin Repeat Containing E3 Ubiquitin Protein Ligase (β-TRCP), which catalyzes its polyubiquitylation. Polyubiquitylated β-catenin, in turn, is degraded by the proteasome. (right part) In the presence of Wnt ligand, the signalosome is assembled and the β-catenin degradation is disrupted, leading to cytoplasmic accumulation. Entry of β-catenin into the nucleus promotes the formation of the enhanceosome to drive the transcription of Wnt target genes [Illustration adapted from Anthony et al. (19)]. **(B)** In our two pancreoblastoma cases. (left part) The CTNNB1 S37C mutation present in case 1 does not allow the β-catenin phosphorylation by CK1this site. (right part) The APC deletion in case 2 does not allow the building of β-catenin destruction complex. Both alterations, CTNNB1 S37C mutation and APC deletion, prevent the β-catenin interaction with E3 ubiquitine ligase and its degradation by the proteasome. The β-catenin accumulation in the cytoplasm leads to its constitutive translocation into the nucleus promoting the formation of the enhanceosome where its binding with TCF results in the constitutive activation of the Wnt signaling pathway.

Activation of the Wnt/β-catenin pathway participates to the tumorigenesis of many organs by regulating the expression of genes involved, for example, in proliferation, cell survival or adhesion. The proteins coded by *CTNNB1* and *APC* are crucial effectors of Wnt signaling. Several alterations in the pathway can cause ligand-independent β-catenin stabilization, which thus contributes to oncogenic β-catenin-regulated transcriptional activity. Many regulators have been identified which control the β-catenin subcellular location, nuclear abundance or transcriptional activity. Figure 4 displays the Wnt/β-catenin pathway in normal tissues (Figure 4A) and in our two cases (Figure 4B). *CTNNB1* exon 3 mutations lead to alterations in the spatial characteristics of the β-catenin protein, decreasing its degradation, and resulting in accumulation in the nucleus (20). Such aberrant nuclear accumulation of β-catenin, hallmark of Wnt/β-catenin activation, is detectable by IHC (16), as observed in our case 1 and the literature. *APC* regulates the turnover of cytosolic β-catenin and is the key effector of the canonical Wnt pathway. Its loss-of-function, either by mutation or by deletion as in our case, reduces the ability to bind to β-catenin, decreasing its degradation, and resulting in accumulation in the nucleus, as observed in our case 2 and the literature. We did not analyse the transcriptional consequences of Wnt pathway activation in our two patients. But, Japanese authors [8] have shown in their pediatric cases that such *CTNNB1* or *APC* mutations resulted in Wnt/β-catenin activation and transcriptional consequences with an expression profile characteristic of early pancreas progenitor-like cells along with upregulation of the R-spondin/LGR5/RNF43 module, as well as overexpression of negative-feedback regulators of Wnt pathway (such as *NOTUM* and *NKD1*) and Wnt pathway effectors (such as *LEF1* and *TCF7*). Of note, the authors identified a concurrent imprinting dysregulation of IGF2, as already reported, although at lower frequency in other pediatric tumors like hepatoblasoma (21) and Wilm's tumor (22); such Wnt/IGF2 coactivation defines a consensus molecular subtype of colorectal cancer, characterized by intestinal stem cell–like phenotype and enriched LGR5 signature (23).

Today no targeted therapy has been clearly demonstrated as efficient for tumors with *CTNNB1* mutation, although everolimus plus letrozole (24) and cabozantinib (25) showed some efficacy in patients with endometrial carcinoma. The German series also reported molecular alterations in FGFR signaling in three out of four patients, leading to the delivery of nindetanib, a tyrosine kinase inhibitor targeting FGFRs 1-3. However, the treatment therapy was stopped after 4 weeks because of clinical deterioration (4). No FGFR alteration was observed in our cases. Clearly, the frequent alteration of the Wnt/β-catenin pathway in the reported cases suggests a role and thus a potential therapeutic interest of targeting this pathway. Clearly functional analyses on pre-clinical models are warranted to confirm such role and the interest for targeting the Wnt/β-catenin pathway. Over the past decades, a number of Wnt inhibitors have been identified, but none of them resulted in inhibition of Wnt signaling *via* direct β-catenin targeting. These disappointing observations, associated with the fact that β-catenin is a transcription factor, raised questions regarding its druggability (26). However, several strategies are ongoing to facilitate the development of new therapeutic agents against β -catenin (26, 27).

In conclusion, we report the clinical outcome and molecular profile of two additional cases of sporadic metastatic pancreatoblastoma in young adults. Both patients benefited from systemic FOLFIRINOX regimen that provided a long-lasting minor response in one case and an ongoing partial response in the second one. Exome sequencing showed a *CTNNB1* mutation (exon 3) in the first case and a deletion involving *APC* in the second case, likely responsible for activation of Wnt/β-catenin signaling pathway. These alterations are frequent in pediatric and adult cases, and can help to differentiate acinar cell carcinomas, solid-pseudopapillary neoplasms and pancreatoblastoma from pancreatic ductal adenocarcinoma. Unfortunately, until now, no therapy targeting such alterations is available. Molecular analysis of larger series is warranted.

## Data Availability Statement

The original contributions presented in the study are included in the article/supplementary material, further inquiries can be directed to the corresponding author/s.

## Ethics Statement

Written informed consent was obtained from the individual(s) for the publication of any potentially identifiable images or data included in this article.

## Author Contributions

J-LR, SO-T, and FB: writing of the manuscript. J-LR, SO-T, TL, and LB: cares of the patients. FP and M-FH: pathology. FB, JA, AG, and MC: genomics analysis. All authors: corrections, modifications, and final acceptation.

## Conflict of Interest

The authors declare that the research was conducted in the absenceof any commercial or financial relationships that could be construed as a potential conflict of interest.
